# Identifying FDA-approved drugs with multimodal properties against COVID-19 using a data-driven approach and a lung organoid model of SARS-CoV-2 entry

**DOI:** 10.1186/s10020-021-00356-6

**Published:** 2021-09-09

**Authors:** Rodrigo R. R. Duarte, Dennis C. Copertino, Luis P. Iñiguez, Jez L. Marston, Yaron Bram, Yuling Han, Robert E. Schwartz, Shuibing Chen, Douglas F. Nixon, Timothy R. Powell

**Affiliations:** 1 Division of Infectious Diseases, Department of Medicine, Weill Cornell Medicine, Cornell University, Belfer Research Building, 5th floor, 413 E. 69th St., New York, NY 10021 USA; 2 Social, Genetic & Developmental Psychiatry Centre, Institute of Psychiatry, Psychology & Neuroscience, King’s College London, London, UK; 3 Division of Gastroenterology and Hepatology, Department of Medicine, Weill Cornell Medicine, Cornell University, New York, NY USA; 4 Department of Surgery, Weill Cornell Medicine, Cornell University, New York, NY USA; 5 Department of Physiology, Biophysics and Systems Biology, Weill Cornell Medicine, Cornell University, New York, NY USA

**Keywords:** Drug repositioning, Lung organoids, Pandemic, COVID-19, Connectivity mapping, Chemoinformatics, Molecular docking, Drug screening, Drug testing, Atorvastatin

## Abstract

**Background:**

Vaccination programs have been launched worldwide to halt the spread of COVID-19. However, the identification of existing, safe compounds with combined treatment and prophylactic properties would be beneficial to individuals who are waiting to be vaccinated, particularly in less economically developed countries, where vaccine availability may be initially limited.

**Methods:**

We used a data-driven approach, combining results from the screening of a large transcriptomic database (L1000) and molecular docking analyses, with in vitro tests using a lung organoid model of SARS-CoV-2 entry, to identify drugs with putative multimodal properties against COVID-19.

**Results:**

Out of thousands of FDA-approved drugs considered, we observed that atorvastatin was the most promising candidate, as its effects negatively correlated with the transcriptional changes associated with infection. Atorvastatin was further predicted to bind to SARS-CoV-2’s main protease and RNA-dependent RNA polymerase, and was shown to inhibit viral entry in our lung organoid model.

**Conclusions:**

Small clinical studies reported that general statin use, and specifically, atorvastatin use, are associated with protective effects against COVID-19. Our study corroborrates these findings and supports the investigation of atorvastatin in larger clinical studies. Ultimately, our framework demonstrates one promising way to fast-track the identification of compounds for COVID-19, which could similarly be applied when tackling future pandemics.

**Supplementary Information:**

The online version contains supplementary material available at 10.1186/s10020-021-00356-6.

## Background

Coronavirus Disease 2019 (COVID-19), caused by the severe acute respiratory syndrome coronavirus type 2 (SARS-CoV-2), instigated the current global public health crisis that has put our society on hold. While most individuals recover successfully, COVID-19 is associated with an alarming mortality rate of 1.5–15.2%, which varies across nations (Baud et al. 2020), and depends on regional healthcare resources (Ji et al. 2020), and patient characteristics such as comorbidities (Guan et al. 2020a), age (Onder et al. 2020), and sex (Guan et al. 2020b). Although successful vaccination programs are underway, it may take longer for less economically developed countries to widely distribute these vaccines (Yamey et al. 2020; Fidler et al. 2011), meaning many lives remain at risk. Additionally, while drugs like remdesivir (Beigel et al. 2020) and dexamethasone (The RECOVERY Collaborative Group 2021) have been useful for treating severe cases, they are not useful for mild cases (e.g., remdesivir (Beigel et al. 2020)) or for prophylaxis (e.g., prolonged exposure to dexamethasone is associated with increased risk of infections, weight gain, metabolic abnormalities, and osteoporosis (WHO 2020)). Therefore, identifying safe drugs that have the combined potential to prevent and treat COVID-19 could alleviate the burden of COVID-19 to individuals, society, and to our health care systems.

**Fig. 1:**
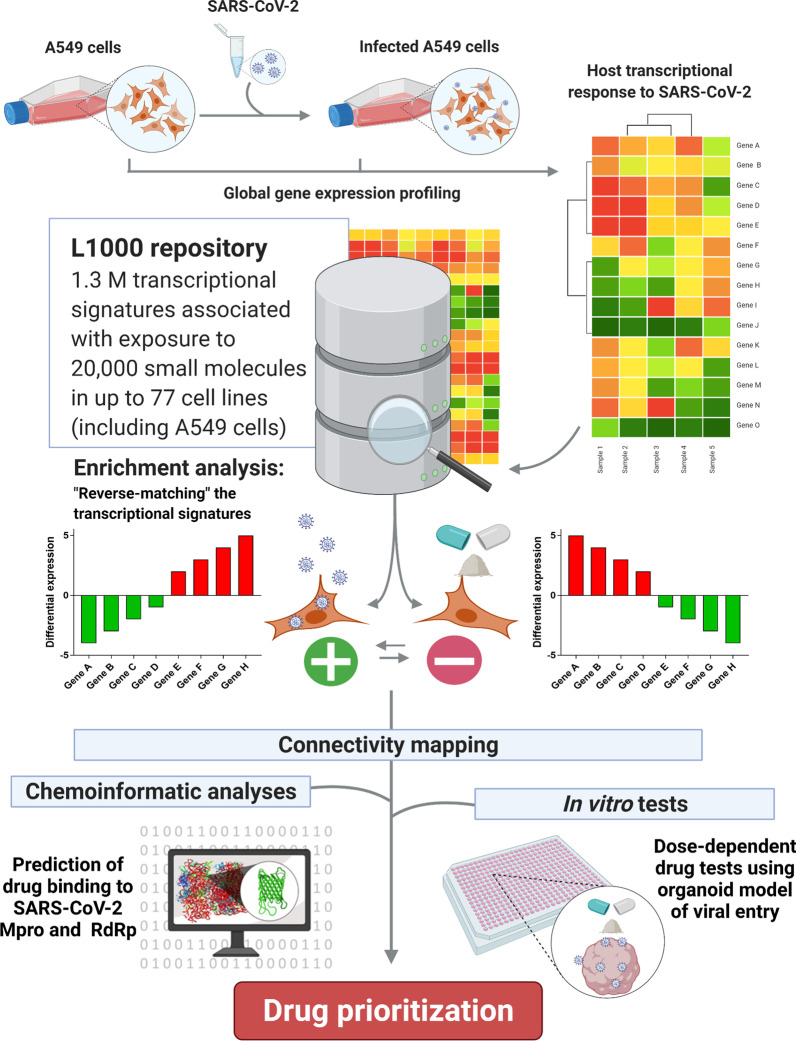
Our analysis strategy. We used a connectivity mapping approach in combination with chemoinformatic analyses and in vitro tests using a lung organoid model, to investigate drugs with putative multimodal effects against SARS-CoV-2/COVID-19. Created with Biorender.com

One approach to identify existing drugs with treatment potential is ‘connectivity mapping’, a data-driven method that has been used to identify compounds with repurposing potential in diverse areas of medicine (Keenan et al. 2019). Successful examples of repurposed drugs identified via connectivity mapping include: ursolic acid for treating muscular atrophy (Kunkel et al. 2011), chlorpromazine for the treatment of hepatocellular carcinoma (Lee et al. 2015), and celastrol for the treatment of obesity (Liu et al. 2015). As part of the connectivity mapping pipeline, a disease-associated transcriptional signature (e.g., observed in a model of SARS-CoV-2 infection) is queried against a repository of transcriptional signatures, like the L1000 database (Subramanian et al. 2017). In its current version, this repository contains 1.3 million transcriptional profiles associated with the effect of 27,927 perturbagens, including drugs, gene knockdowns and knock-ins, tested in up to 77 cell lines. After a series of enrichment analyses, drugs with repurposing potential are selected based on their ability to elicit the reverse transcriptional signature that was originally queried (see Fig. 1 for a visual representation of the connectivity mapping approach). The usefulness of these drugs can then be validated in subsequent computational and in vitro studies.

When identifying compounds with prophylactic and treatment potential for viral infections, there are chemoinformatic methods that predict which drugs may bind to viral enzymes playing crucial roles in virus replication, such as the main protease (Mpro) or the RNA-dependent RNA polymerase (RdRp), potentially blocking their function (Singh et al. 2020). In addition, it is possible to investigate whether specific drugs are likely to work in biological systems by testing their ability to block parts of the viral cycle in in vitro models of infection (Han et al. 2020; Froggatt et al. 2020; Zhao et al. 2021). For example, Han et al. (2020) generated a sophisticated COVID-19 lung organoid model utilizing SARS-CoV-2-Spike pseudovirus particles, to screen for compounds that could block viral entry. Here, we sought to identify FDA-approved drugs with potential treatment and prophylactic properties for use against COVID-19, using convergent evidence from connectivity mapping and molecular docking analyses, collectively with an in vitro lung organoid model of SARS-CoV-2.

## Methods

### Connectivity mapping

Our analysis strategy is depicted in Fig. 1. Briefly, we used CMap v1.1.1.43, dataset v1.1.1.2, accessed via https://clue.io (Subramanian et al. 2017), to identify drugs with repurposing potential for COVID-19, based on the transcriptional signature of A549 cells transfected with an *ACE2* vector (to enable viral entry and replication) and subsequently infected with SARS-CoV-2, as described in Blanco-Melo et al. (2020). We selected genes that were up- and down-regulated after infection under the false discovery rate (FDR) of 5%, as input for CMap, to search for compounds in the L1000 database that elicited the reverse of the transcriptional signature associated with infection. This platform calculates connectivity scores for each drug based on the observed enrichment scores in the queried gene lists relative to the transcriptional signatures contained in the L1000 reference database. The score incorporates a nominal p-value calculated based on the comparison between the query and reference signatures relative to a null distribution of random queries, using the Kolmogorov–Smirnov enrichment statistic, which is then corrected for multiple testing using the FDR method. These values are converted to *tau* values (τ) by comparing the resulting value with reference signature queries in the L1000 repository (Subramanian et al. 2017; Subramanian et al. 2005). The authors suggest that drugs with τ < − 90.00 are those more likely to reverse the transcriptional signature queried, and conversely, those with τ > 90.00 are more likely to mimic it. We were interested in drugs with potential to reverse the mRNA signature associated with SARS-CoV-2 infection (i.e., those with τ < − 90.00), particularly in A549 cells, thus matching the same cell line used to model infection by Blanco-Melo et al*.* (2020) and avoiding potential biases due to unmatched cell types. We considered small compounds with τ < − 90.00, that were FDA-approved, according to Corsello et al*.* (2017), as hopeful candidates for repurposing and, therefore, considered only those for downstream analyses.

### Molecular docking analyses

Amongst selected candidates identified through the connectivity mapping analyses, we sought to infer whether they were predicted to bind to key SARS-CoV-2 enzymes using a chemoinformatic approach (as previously, e.g., (Copertino et al. 2021a)), to provide an indication of potential multi-modal effects against COVID-19. We performed molecular docking simulations on the RNA-dependent RNA polymerase (RdRp) (Protein Data Bank ID: 6M71), and the main protease (Mpro) (PDB ID: 6Y2E) of SARS-CoV-2, using default settings in the Protein–Ligand ANT System (PLANTS) (Korb et al. 2009), as described elsewhere (Copertino et al. 2021b). The ligand docking sites were specified, respectively, as the catalytic sites determined by Zhang et al. (2020a) (Gln189) and Gao et al. (2020) (Asp623), using an estimated radius of 10 Å around the specified residues. The resulting protein–ligand scores (PLANTS scores), calculated using the CHEMPLP algorithm, reflect the energy change when ligands and proteins come together, with values less than − 80.00 suggesting effective ligand–protein interactions. PLANTS files were converted to Mae files in PyMol 2.3 (The PyMOL Molecular Graphics System, Version 2.0 Schrödinger, LLC), and loaded into Maestro (Bowers et al. 2006) for visualization.

### Cell lines

We used the lung organoid model of SARS-CoV-2 infection described in Han et al. (2020) to identify drugs with the ability to block viral entry. Human embryonic stem cells (hESCs) RUES2 (WiCell, Madison, Wisconsin, United States) were maintained on 1% Matrigel-coated six-well plates in StemFlex medium (Gibco) at 37 °C with 5% CO_2_ culture condition. The medium was changed daily. When hESCs reached ~ 90% confluence, the cells were passaged at 1:6–1:10 with ReLeSR (Stem Cell Technology). To produce viral particles for the viral entry assay, we used human fetal kidney cells HEK293T. These cells were obtained from ATCC and cultured in DMEM supplemented with 10% fetal bovine serum, 100 I.U./mL penicillin and 100 μg/mL streptomycin, and incubated at 37 °C with 5% CO_2_/air.

### Lung organoids

RUES2 hESCs were differentiated into endodermal cells, and subsequently into lung cells. Differentiation to endodermal cells was performed in serum-free differentiation (SFD) medium of DMEM/F12 (3:1) (Life Technologies) supplemented with N2 (Life Technologies), B27 (Life Technologies), 50 μg/mL ascorbic acid, 2 mM Glutamax (Gibco), 0.4 μM monothioglycerol, 0.05% BSA, with incubation at 37 °C, with 5% CO_2_, 5% O_2_, and 95% N_2_. These cells were re-suspended in endoderm induction medium containing 10 μM Y-27632, 0.5 ng/mL human BMP4 (R&D Systems), 2.5 ng/mL human bFGF, 100 ng/mL human Activin A (R&D Systems), for 72–76 h, until the formation of endodermal cells. Subsequently, endoderm bodies were dissociated using a 0.05% Trypsin 0.02% EDTA mixture, re-plated, cultured in SFD medium supplemented with 1.5 μM dorsomorphin dihydrochloride (R&D Systems) and 10 μM SB431542 (R&D Systems) for 36 h, and then incubated for another 36 h with 10 μM SB431542 and 1 μM IWP2 (R&D Systems). The resulting cells were treated with 3 μM CHIR99021 (CHIR, Stem-RD), 10 ng/mL human FGF10, 10 ng/mL human KGF, 10 ng/mL human BMP4 and 50–60 nM all-trans retinoic acid (ATRA), in SFD medium. On days 10–15, cultures were incubated with 5% CO_2_/air. On days 15–16, lung field progenitor cells were re-plated and incubated in SFD media supplemented with 3 μM CHIR99021, 10 ng/mL human FGF10, 10 ng/mL human FGF7, 10 ng/mL human BMP4, and 50 nM ATRA. On days 16–25, cultures were maintained in SFD media containing 3 μM CHIR99021, 10 ng/mL human FGF10, 10 ng/mL human KGF. In days 25–55, we differentiated mature lung cells onto 3D cultures by re-plating and embedding cells in 90% Matrigel in SFD media supplemented with 3 μM CHIR99021, 10 ng/mL human FGF10, 10 ng/mL human KGF, 50 nM Dexamethasone, 0.1 mM 8-bromo-cAMP (Sigma Aldrich), and 0.1 mM IBMX (3,7-dihydro-1-methyl-3-(2-methylpropyl)-1H-purine-2,6-dione, Sigma Aldrich). The resulting cells show high similarity to adult human lung AT2 cells, as described previously by Han et al. (2020).

### SARS-CoV-2-entry viruses

Recombinant Indiana vesiculovirus (rVSV) expressing the SARS-CoV-2 spike protein were generated as previously described (Han et al. 2020; Yang et al. 2020). Briefly, HEK293T cells were grown to 80% confluency and subsequently transfected with pCMV3-SARS-CoV-2-spike (kindly provided by Dr. Peihui Wang, Shandong University, China) using FuGENE 6 (Promega), and incubated overnight at 37 °C with 5% CO_2_. On the next day, the medium was removed and VSV-G pseudo-typed ΔG-luciferase (G*ΔG-luciferase, Kerafast) was used to infect the cells in DMEM (MOI = 3.0) for 1 h, before washing the cells with DPBS three times. DMEM supplemented with anti-VSV-G antibody (I1, mouse hybridoma supernatant from CRL2700, ATCC) was added to the infected cells and incubated overnight. On the next day, the supernatant was collected and centrifuged at 300×*g* for 10 min, and aliquots were stored at − 80 °C.

### Drug tests in vitro

The lung organoids were dissociated using TrypLE for 10 min at 37 °C, and cells were re-plated into 10% Matrigel-coated 384-well plates at a density of 10,000 cells/40 µL medium per well. Six hours after plating, compounds were added. Drugs were purchased from Sigma, and were diluted in DMSO. The lung organoids were infected with SARS-CoV-2-entry virus (MOI = 0.01) and centrifuged at 1200×*g* for 1 h. After incubation at 37 °C with 5% CO_2_ for 24 h, the cells were harvested for subsequent analysis using the luciferase assay (Luciferase Assay System protocol E1501, Promega) or to quantify cell survival using Cell-Titer Glo (Promega). We tested three drugs (trifluoperazine, atorvastatin, and raloxifene) for a dose-dependent inhibition of luciferase activity, using three concentrations (10 µM, 33 µM, 100 µM), alongside doxycycline which was used as a positive control (Gendrot et al. 2020). Trifluoperazine, atorvastatin, and raloxifene were selected based on the connectivity mapping results, their predicted ability to bind to SARS-CoV-2 Mpro and RdRp enzymes, and their availability in our lab. For atorvastatin and doxycycline, we calculated the half maximal inhibitory concentration (IC50) required to inhibit viral replication, and the half maximal cytotoxic concentration (CC50), as an indication of cell toxicity, using a ten-point curve of 1:3 dilutions (highest concentration: 100 µM; lowest concentration: 5 nM), normalizing the luciferase activity to the lowest concentration tested. The efficacy and cytotoxicity curves were calculated in Prism GraphPad 7.0.

### Transcriptional effects of atorvastatin in A549 cells

We retrieved the genes differentially expressed in A549 cells treated with 10 µM atorvastatin according to the L1000 repository, by accessing https://clue.io. Genes were considered up- or downregulated if their Z scores were consistently above or below zero, respectively, across the 6- and 24-h treatments. P-values were corrected using the FDR method, and only genes with FDR < 0.05 were considered significant. Gene ontology (GO) analysis was performed using Webgestalt (Liao et al. 2019) to identify non-redundant GO terms (biological processes, cellular components, or molecular functions) associated with treatment. Only terms with FDR < 0.05 were considered significant. Plots were generated in Webgestalt. To investigate whether there was an enrichment of genes downregulated by atorvastatin in the genes upregulated in the model of infection (Blanco-Melo et al. 2020), we performed a Fisher’s exact test in R using the *GeneOverlap* library (Shen et al. 2021).

## Results

### Connectivity mapping reveals drugs with repurposing potential for treating COVID-19

We used the transcriptional signature associated with SARS-CoV-2 infection in A549 cells, according to Blanco-Melo et al*.* (2020), to search for drugs with repurposing potential for treating COVID-19 (Additional file 1: Table S1). A549 cells originate from a human alveolar adenocarcinoma sample, and represent a useful in vitro model of the lung epithelium (e.g., Gminski et al. (2010)). Our analysis revealed 76 drugs with potential to reverse the transcriptional signature associated with SARS-CoV-2 infection in A549 cells specifically (τ < − 90.00). Of the 76 drugs with τ < − 90.00, we observed that 26 were small compounds which were already FDA approved, according to Corsello et al. (2017). These included the drug used for treating hypercholesterolemia and preventing cardiac disease, atorvastatin (τ = − 96.7), the antipsychotic drugs trifluoperazine (τ = − 97.9) and flupentixol (τ = − 95.9), and the estrogen receptor modulator raloxifene, used for treating osteoporosis (τ = − 96.1), amongst others (Additional file 1: Table S2).

### Molecular docking analyses

**Fig. 2:**
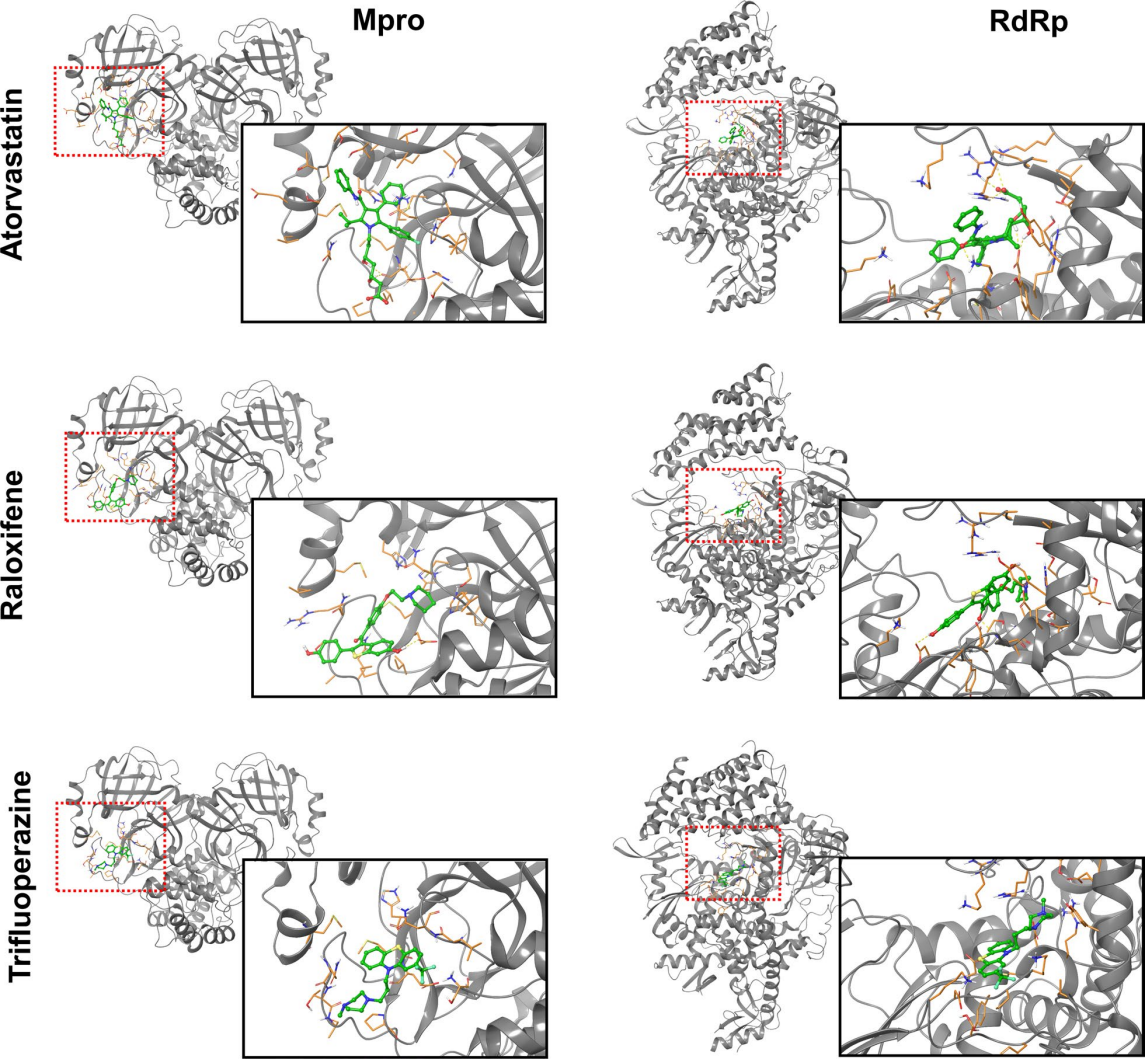
Visualization of the predicted interactions between atorvastatin, raloxifene and trifluoperazine, and the SARS-CoV-2 Mpro (left panel) and RdRp (right panel)

From the subset of drugs identified in the connectivity mapping analysis, we performed molecular docking analyses to infer their predicted ability to bind to the catalytic sites of the SARS-CoV-2 Mpro and RdRp. We hypothesized that, if the effect of these compounds negatively correlated with the transcriptional signature associated with infection (according to the connectivity mapping analysis), and these drugs were additionally predicted to bind to, and potentially block, viral Mpro and RdRp, they would be more likely to exert multimodal effects against COVID-19. We found that atorvastatin, flupentixol, raloxifene, and trifluoperazine, were predicted to bind to both Mpro and RdRp (PLANTS scores < − 80.00), thus corroborating putative multimodal actions (see Table 1 for full results summary, Fig. 2 for protein–ligand interactions visualization, and Additional file 1: Table S3 for details of the chemical structures analyzed).

**Table 1:** Results summary

Drug name	Established function	Connectivity score (τ)	RdRp docking (PLANTS score)	MPro docking (PLANTS score)	Effect in vitro
Reserpine	Vesicular monoamine transporter inhibitor	− **99.94**	N/A	−** 80.72**	–
Hydroquinidine	Antiarrhythmic	**− 99.44**	− 68.79	− 79.05	–
Fluoxetine	Selective serotonin reuptake inhibitor (SSRI)	**− 99.20**	− 77.63	−** 81.14**	–
Maprotiline	Norepinephrine reuptake inhibitor	**− 99.10**	− 72.42	− 76.15	–
Tamoxifen	Estrogen receptor antagonist	**− 98.87**	− 57.40	− 63.05	–
Dextromethorphan	Glutamate receptor antagonist	**− 98.82**	N/A	− 62.08	–
Phensuximide	Succinimide antiepileptic	**− 98.77**	N/A	− 66.02	–
Sulpiride	Dopamine receptor antagonist	**− 98.58**	− 72.36	− 64.34	–
Metformin	Insulin sensitizer	**− 98.27**	− 32.82	− 31.63	–
Trifluoperazine	Dopamine receptor antagonist	**− 97.90**	−** 84.29**	−** 84.43**	N
Sunitinib	FLT3 inhibitor	**− 96.82**	− 73.52	−** 80.93**	–
Irsogladine	Phosphodiesterase inhibitor	**− 96.78**	− 60.14	− 61.59	–
Atorvastatin	HMGCR inhibitor	**− 96.65**	−** 89.22**	−** 82.17**	**Y**
Raloxifene	Estrogen receptor antagonist	**− 96.09**	−** 84.32**	−** 87.57**	N
Flupentixol	Dopamine receptor antagonist	**− 95.90**	−** 91.70**	−** 91.82**	N/A
Alprenolol	Adrenergic receptor antagonist	**− 95.62**	− 68.19	− 68.69	–
Bosutinib	ABL inhibitor	**− 94.89**	− 76.61	− 76.87	–
Buflomedil	Adrenergic receptor antagonist	**− 94.76**	− 63.00	− 67.89	–
Guanfacine	Adrenergic receptor agonist	**− 94.09**	− 59.95	− 61.08	–
Epirizole	Cyclooxygenase inhibitor	**− 92.86**	− 61.37	− 67.36	–
Diloxanide	Protein synthesis inhibitor	**− 92.75**	− 58.14	− 58.46	–
Clomipramine	Serotonin transporter inhibitor (SERT)	**− 92.70**	− 66.44	− 78.01	–
Sulfacetamide	PABA antagonist	**− 92.70**	− 55.60	− 54.92	–
Norethindrone	Progesterone receptor agonist	**− 92.69**	N/A	− 66.15	–
Doxycycline	Bacterial 30S ribosomal subunit inhibitor	**− 92.29**	− 72.86	− 74.47	Y*
Butoconazole	Bacterial cell wall synthesis inhibitor	**− 92.07**	− 77.24	−** 82.92**	–

This table includes a compilation of the results obtained from the connectivity mapping analysis, the chemoinformatic analyses, and the in vitro drug tests using the lung organoid model. The table is ordered according to each drug’s connectivity mapping score. Bold values indicate those that survive our cut-offs (τ < − 90.00 for the connectivity mapping analysis; PLANTS score < − 80.00 for the chemoinformatic analyses), or whether a dose-dependent effect was observed in the corresponding in vitro test (yes/no). N/A: the drug was associated with a very low PLANTS score, or the drug was unavailable for in vitro testing (i.e., flupentixol). **Y**: the drug was tested in vitro and showed a promising inhibitory effect. Y*: the drug was the positive control (doxycycline) and showed an inhibitory effect in vitro. N: the drug was tested in vitro but did not show an inhibitory effect in the lung organoid model. Dashes ('–') represent drugs that were not tested in vitro

### Atorvastatin blocks SARS-CoV-2 entry in lung organoids

**Fig. 3:**
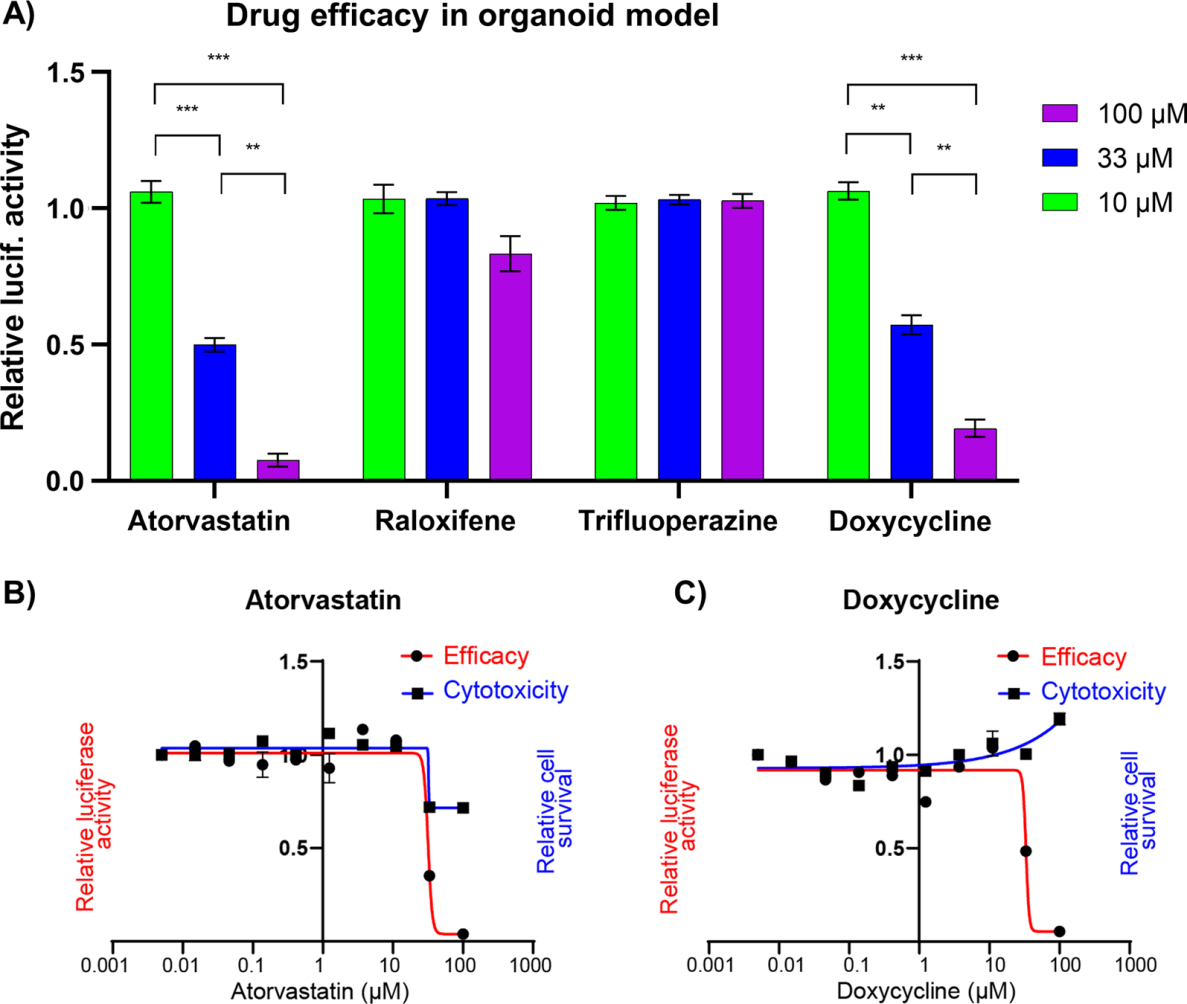
The dose-dependent drug screening of three drugs whose transcriptional profiles correlate negatively with the host transcriptional signature associated with SARS-CoV-2 infection in the connectivity mapping analysis, which were further selected based on their predicted ability to bind to SARS-CoV-2 Mpro and RdRp. **A** Atorvastatin was the only drug that showed a dose-dependent reduction in viral entry in the SARS-CoV-2 lung organoid model, alongside our positive control (doxycycline). The bar plot shows the relative luciferase activity of multiple candidate drugs at 100 μM, 33 μM and 10 μM. The data was normalized to DMSO treated control. Data is presented as mean ± SEM. Two-way ANOVA, **P < 0.01, ***P < 0.001. **B** The dose-dependent efficacy curves of atorvastatin, and **C** doxycycline, suggest that these drugs inhibit viral entry in the SARS-CoV-2 lung organoid model. Their maximum half cytotoxicity concentrations are above the tested range. The data was normalized to DMSO-treated control. Data is presented as mean ± SEM

We investigated the efficacy of these drugs to block SARS-CoV-2 entry using a lung organoid model of infection. Of the four drug candidates from the connectivity mapping analysis that were further predicted to bind to Mpro and RdRp, we tested three which were readily available within our lab (atorvastatin, trifluoperazine, and raloxifene), alongside a drug which is known to inhibit SARS-CoV-2 entry in vitro (doxycycline), as our positive control (Gendrot et al. 2020). The drugs were incubated with lung organoids infected with a vesicular stomatitis ΔG-luciferase virus pseudo-typed with the SARS-CoV-2 spike protein (SARS-CoV-2-entry virus) (Whitt 2010; Nie et al. 2020), and viral entry was assessed by quantification of the luciferase signal, as described in Han et al. (2020). Specifically, the SARS-CoV-2-entry virus generates a luciferase signal in infected cells, unless the drugs are able to block viral entry (e.g., by blocking the virus’ spike protein, or the host’s ACE2 receptor, or even by changing the levels of this receptor). We observed that atorvastatin was associated with reduced luciferase signal in a dose-dependent manner (Fig. 3A; two-way ANOVA, F_(2,4)_ = 290.1, P < 0.0001; Tukey post hoc tests: 100 µM vs. 33 µM, P = 0.0011; 100 µM vs. 10 µM, P < 0.0001; 33 µM vs. 10 µM, P = 0.0004), whereas we did not observe an effect associated with trifluoperazine or raloxifene (P > 0.05). The same dose-dependent reduction in luciferase activity was observed in our positive control, i.e. cultures treated with doxycycline (two-way ANOVA, F_(2,4)_ = 171.2, P = 0.0001; Tukey post hoc tests: 100 µM vs. 33 µM, P = 0.0028; 100 µM vs. 10 µM, P = 0.0001; 33 µM vs. 10 µM, P = 0.0011). To understand whether the inhibitory effects associated with atorvastatin and doxycycline were truly driven by inhibition of viral entry, rather than via a reduction in cell viability, we calculated the IC50 and CC50 associated with these drugs using a ten-point curve of 1:3 dilutions, from 100 µM to 5 nM. We were unable to calculate CC50 for either drug within this range, suggesting that their half maximum cytotoxic concentrations in the lung organoids were quite high, above 100 µM (i.e., the drugs were safe for our model even at the highest concentrations tested). In terms of inhibition of viral entry, we observed that atorvastatin and doxycycline were associated with IC50 values of 31.65 μM and 33.31 μM, respectively (Fig. 3B, C). These concentrations are at least a magnitude higher relative to the top compounds identified in a drug screening performed using the same experimental setup (imatinib: IC50 = 4.86 μM; mycophenolic acid: IC50 = 0.15 μM; quinacrine dihydrochloride, IC50 = 2.83 μM; see Han et al. (2020)). However, atorvastatin is still a relevant drug candidate as it is more commonly used than these other drugs, so we understand its clinical properties better. Furthermore, atorvastatin is also predicted to act against SARS-CoV-2 on multiple levels (i.e., partly reversing the host transcriptional response associated with infection, putatively blocking Mpro and RdRp, and also blocking viral entry in vitro), potentially conferring protection against multiple risk mechanisms associated with COVID-19.

### Atorvastatin is associated with reduced expression of several immune genes

**Fig. 4:**
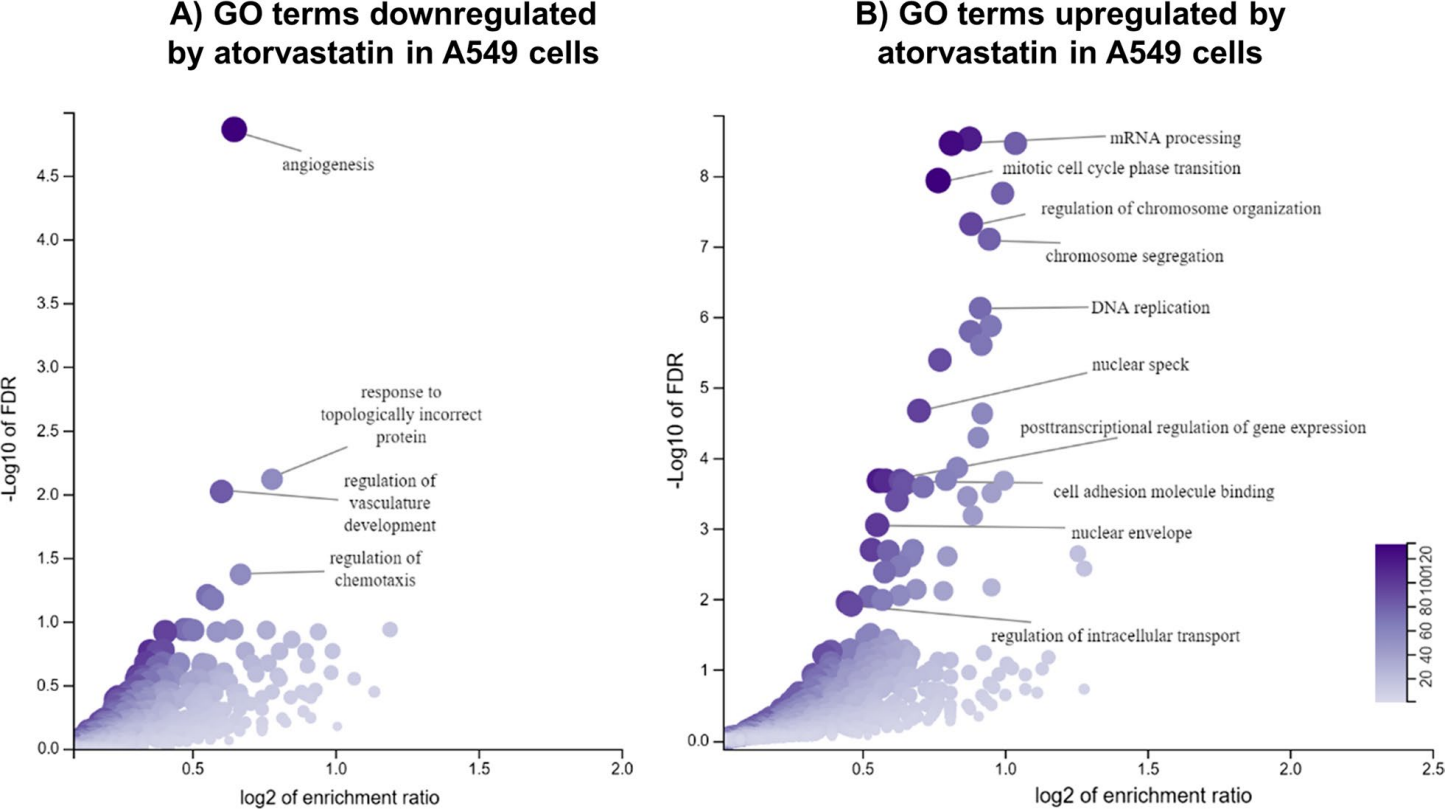
Gene ontology analysis of the genes differentially regulated in A549 cells upon treatment with atorvastatin, based on transcriptomic data from the L1000 repository. Volcano plots show the GO terms enriched within the **A** downregulated and **B** upregulated genes after treatment with this drug. The Y axis shows significance in the − log10 scale, and the X axis, the enrichment ratio for each GO term (represented as dots in the graph)

To further understand how atorvastatin may be beneficial against SARS-CoV-2, we retrieved from the L1000 repository the genes concordantly differentially regulated in A549 cells after 6- and 24-h treatments. We observed that genes positively regulated by atorvastatin (FDR < 0.05) were enriched for gene ontology (GO) terms related to DNA replication and cell cycle regulation (Fig. 4A), consistent with previous work suggesting a role for statins in these processes (Gbelcová et al. 2017). Downregulated genes (FDR < 0.05), in turn, were enriched for GO terms related to angiogenesis and vasculature development (Fig. 4B), which have been previously reported to be modulated by atorvastatin in human umbilical endothelial cells (Dulak et al. 2005).

In relation to its protective role against SARS-CoV-2, we observed that the atorvastatin treatment was associated with the downregulation of many immune genes that are upregulated in the A549 infection model (Blanco-Melo et al. 2020). These include genes related to interferon response (*IFI16, IFI27, IFI44, IFI6, IFIH1, IFIT2, IFIT3, IRF7*), production of chemokines (*CCL20, CXCL2, CXCL8*), and complement activation (*C1R, C3*). Indeed, we observed that the genes upregulated during infection (FDR < 0.05; N = 100) were significantly enriched for genes downregulated by atorvastatin (FDR < 0.05, N = 12,328) (Fisher’s exact test, P = 0.03, number of genes in the intersection = 28, odds ratio = 1.6). It is plausible that the reduction of certain inflammatory markers by atorvastatin may help the immune system manage the cytokine storm typically associated with severe cases (Zhang et al. 2020b). Ultimately, these data corroborate a multi-modal mechanism through which atorvastatin may be beneficial for tackling COVID-19.

## Discussion

Here, we attempted to fast-track the identification of drugs with multimodal actions against COVID-19 using a combined in silico and experimental screening strategy. We used connectivity mapping and the L1000 repository of transcriptional signatures (Subramanian et al. 2017) to identify FDA-approved drugs that could reverse the mRNA effects associated with SARS-CoV-2 infection in A549 lung cells (Blanco-Melo et al. 2020). To identify drugs with multimodal actions against SARS-CoV-2, we performed chemoinformatic analyses to infer whether drug candidates identified from our connectivity mapping approach were also predicted to bind to the SARS-CoV-2 Mpro and RdRp. The resulting candidates were then tested in vitro for their ability to inhibit viral entry in a SARS-CoV-2 lung organoid model (Han et al. 2020), further supporting putative multi-modal effects against COVID-19.

We observed that atorvastatin, which is used for reducing circulating levels of cholesterol and cardiovascular disease prevention (Sever et al. 2003), met all these criteria. Statins in general are known for having anti-inflammatory effects (Schönbeck and Libby 2004) and for suppressing viral-induced interferon-gamma signaling in human PBMCs (Neurauter et al. 2003). Interestingly, a retrospective study that analyzed 5281 COVID-negative controls and 170 patients hospitalized for COVID-19, showed that general statin use 30 days prior to hospital admission was associated with faster recovery and decreased risk of severe COVID-19 (Daniels et al. 2020). A larger retrospective study, in turn, analyzed 13,981 COVID-19-positive individuals, including 1219 who received statins, and found that general statin use was associated with a reduced hazard ratio (HR = 0.58) at a 28-day follow-up (Zhang et al. 2020c). Similarly, another study analyzed a cohort of 10,541 patients, of which 42% used statins, and observed that statin use was associated with reduced risk of death (adjusted odds ratio = 0.59). More specifically, however, Rodriguez-Nava et al*.* (2020) analyzed a retrospective cohort of 87 SARS-CoV-2-positive patients in intensive care, and found that 40 mg/day atorvastatin was associated with reduced mortality, and a significantly lower hazard ratio (HR = 0.38). Atorvastatin was also effective at preventing the infection of Vero cells infected with SARS-CoV-2 (2019-nCoV/USA-WA1/2020), whereby 5 µM atorvastatin decreased the viral load 1.6-fold in vitro, relative to the vehicle-treated control (Risner et al. 2020). Using the same cell line, another group found that the protective effects of atorvastatin occur both pre- and post-infection (Zapata-Cardona et al. 2021), thus corroborating a model in which atorvastatin may help in prevention and treatment of severe COVID-19.

Atorvastatin is the most widely prescribed statin in the United States, with approximately 80.7 million prescriptions made in 2014 alone (IMS 2015). In addition, atorvastatin is well-tolerated in patients over the age of 65 in dosages up to 80 mg/day, corroborating its safety. Although it remains unclear whether atorvastatin taken orally may become available in lung tissue (Zeki and Elbadawi-Sidhu 2018), this drug is hypothesized to reach the lungs after oral delivery since it is lipophilic, and therefore more likely to cross cell membranes through passive diffusion (McKenney et al. 2009). Ultimately, large randomized-controlled clinical trials would be required to investigate the effects of this drug for treating and preventing COVID-19, and the optimal dosages.

There are limitations to our study that should be acknowledged. For example, it is likely that the transcriptional signature associated with infection depends on length of exposure and cell model used (viral strain and cell type). As such, future connectivity mapping analyses that consider different cell models, or the long-term effects of infection, may yield additional insights. In addition, the chemoinformatic analyses may not necessarily identify true ligand–protein interactions, and therefore crystallography studies or antibody neutralization studies could provide evidence of such interactions. In addition, although the lung organoid model provides a powerful systems-level approach to screen for compounds with putative protective effects against SARS-CoV-2, it models only the entry step of the SARS-CoV-2 replication cycle, and additional models could be useful to test the efficacy of specific drugs against other steps of the viral cycle (e.g., using a SARS-CoV-2 protease reporter (Froggatt et al. 2020), or an RNA dependent RNA polymerase reporter (Zhao et al. 2021). Finally, while we observed that atorvastatin inhibited viral entry in the lung organoid model, the observed IC50 was quite high. A study found that patients taking atorvastatin 40 mg daily for at least six weeks showed a mean plasma concentration of 3 ng/mL (2.48 nM) (DeGorter et al. 2013), which corresponds to a much lower concentration than the IC50 observed in the lung organoid model. Further work is needed to explore how translatable the acute concentrations used in our in vitro model are to those found in vivo.

## Conclusions

Ultimately, our work attempted to identify drugs with multimodal actions against SARS-CoV-2 infection and COVID-19, suggesting atorvastatin as a plausible candidate. Although preliminary clinical studies corroborate the use of atorvastatin and other statins for treating or preventing severe COVID-19 (Daniels et al. 2020; Zhang et a﻿l. 2020c; Rodriguez-Nava et al. 2020; Tan et al. 2020), further clinical investigation is warranted to confirm efficacies and optimal dosages. This novel framework, which combines computational and experimental research, might help to fast-track the identification of FDA-approved drugs for treating and preventing COVID-19, and may be a useful approach when tackling future pandemics.

## Supplementary Information

**Additional file 1: Table S1.** Genes differentially expressed in lung cancer cells after exposure to SARS-CoV-2. Source: Blanco-Melo et al. (2020). **Table S2.** Connectivity mapping results generated by querying the SARS-CoV-2 transcriptional signature into CMap. **Table S3.** Source of the drug structures utilized in the chemoinformatic analysis testing for interaction with SARS-CoV-2 enzymes.

## Data Availability

The transcriptional signature associated with SARS-CoV-2 infection in A549 cells was obtained from the Blanco-Melo et al*.* (2020) study. The L1000 database was accessed via https://clue.io. The source of the drug structures analyzed in the chemoinformatic analyses is listed in Additional file 1: Table S3.

## Abbreviations

COVID-19: Coronavirus disease 2019
SARS-CoV-2: Severe acute respiratory syndrome coronavirus type 2
HR: Hazard ratio
Mpro: Main protease
RdRp: RNA-dependent RNA polymerase
FDR: False discovery rate
hESCs: Human embryonic stem cells
SFD: Serum-free differentiation medium
IC50: Half maximal inhibitory concentration
CC50: Half maximal cytotoxic concentration
